# Biological Pacemakers

**Published:** 2006-01-01

**Authors:** G Rajesh, Johnson Francis

**Affiliations:** Department of Cardiology, Medical College Hospital, Calicut, Kerala, India

**Keywords:** Gene therapy, Pacemaker current, HCN channels

## Abstract

Genetically engineered pacemakers could be a possible alternative to implantable electronic devices for the treatment of bradyarrhythmias. The strategies include upregulation of beta adrenergic receptors, conversion of myocytes into pacemaker cells and stem cell therapy. Pacemaker activity in adult ventricular myocytes is normally repressed by the inward rectifier potassium current (I_K1_). The I_K1_ current is encoded by the Kir2 gene family. Use of a negative construct that suppresses current when expressed with wild-type Kir2.1 is an experimental approach for genesis of genetic pacemaker. Hyperpolarisation activated cyclic nucleotide gated (HCN) channels which generate If current, the pacemaker current of heart can be delivered to heart by using stem cell therapy approach and viral vectors. The unresolved issues include longevity and stability of pacemaker genes, limitations involved in adenoviral and stem cell therapy and creation of genetic pacemakers which can compete with the electronic units.

## Introduction

Implantable electronic pacemakers remain the treatment of choice for high degree atrioventricular blocks and sinus node dysfunction. The shortcomings of electronic pacemakers include limited battery life, need for lead implantation into heart and lack of response to autonomic and physiologic demands on the heart.  Molecular approaches to the development of a biological pacemaker are a conceptually attractive alternate treatment modality for heart blocks. The approaches attempted to provide such pacemaker function  include up regulation of β_2_ adrenergic receptors [1], down regulation of K+ current I_K1_ [2] and over expression of HCN2 (hyperpolarisation activated cyclic nucleotide gated) channels the molecular correlate of the endogenous cardiac pacemaker current I_f_ [3]. The genetic treatment can be applied to heart by plasmid injection, use of viral constructs or stem cell therapy [4] [5].

## Molecular targets for genesis of biological pacemaker

### β_2_ adrenergic receptors

The sinus node has a higher density of β adrenergic receptors (βAR) compared with surrounding atrium [1]. This density of βAR and its regulation of If current suggest that increases in the density of βAR in the vicinity of the sinus node may lead to an increase in heart rate. The up regulation of β_2_ adrenergic receptors can be achieved by plasmid injection into heart. It was noted that after injection of plasmids in porcine right atrium heart rates were 50% faster than those of controls. One potential limitation of this strategy is that the diseased endogenous cardiac pacemaker mechanisms are left intact and the β_2_ receptor is used as a nonspecific stimulator of heart rate. It can influence other catecholamine sensitive channels also.

### HCN channel and I_f_ current

Action potential of pacing cells is unique in that they have a slow depolarizing phase, rendering them spontaneously active [6]. The depolarization involves interaction between HCN channels and L & T type calcium channels. The modification of these channels is a therapeutic target.

HCN channels generate I_f_ current which contribute to genesis of pacemaker activity. I_f_ channel is activated on membrane hyperpolarisation rather than on depolarization [7]. It has four fold selectivity for K+ than Na+. The typical features of I_f_ current include activation by hyperpolarized membrane potential, conduction of Na+ and K+, modulation by cyclic adenosine monophospate (CAMP) and blockade by cesium (Cs+) [8]. HCN generated current also has the above features. Four different HCN genes have been identified [9]. HCN1 is the most rapidly acting channel, HCN4 the slowest with HCN2 and 3 possessing intermediate kinetics [10]. HCN1, 2 and 4 have been found to express in adult heart, HCN4 being the most highly expressed one in SA node. HCN2 expression was noted in atrium, ventricle and SA node.

HCN can be delivered to heart by adenoviral construct or using stem cells. The nucleic acids delivered by adenoviruses do not integrate into genome as they are episomal. Stem cell therapy may be more promising than viral strategy. The approach using HCN may be less problematic and proarrythmic as it incorporates the endogenous pacemaker channel gene, which selectively activates only during diastole [14].

### Inward Rectifier Potassium Current (I_K1_)

I_K1_ and other background K+ selective currents contribute to action potential depolarization and establish diastolic resting membrane potential. Down regulation of the background K+ current I_K1_ is one of the approaches attempted to provide pacemaker function. Genetic suppression of I_K1_ can converts quiescent myocytes into pacemaker cells.

I_K1_ is the classical inward rectifier potassium current. Inwardly rectifying K+ channels (Kir) are responsible for stabilizing the resting membrane potential. Inward rectification is a phenomenon in which conductance of a Kir channel increases with hyperpolarisation but decreases with depolarization. Rectification in Kir channels results from voltage dependent channel block by intracellular cations [12]. IK1 is absent or poorly expressed in sinus and AV nodal cells. Native I_K1_ in human ventricular myocytes is reduced by adrenergic receptor stimulation.

It was observed that a dominant negative strategy to reduce I_K1_, which usually maintain ventricular myocytes at negative membrane potentials, induced spontaneous impulse initiation in guinea pig heart. The inward rectifier potassium current is encoded by Kir2 gene family. Replacement of 3 amino acid residues in the pore structure of Kir2.1 creates a dominant negative construct [12]. Downregulation of I_K1_ removes an important determinant of repolarisation leading to prolonged repolarisation in cells lacking this current [13]. This may result in excessive dispersion of repolarisation leading to theoretical risk of proarrhythmia.

## Stem cell therapy

Human embryonic stem cells can be used to create pacemakers or adult mesenchymal stem cells may be used as platforms for delivery of pacemaker genes to myocardium. The advantage of these cells includes their ability to make functional gap junctions and generate spontaneous rhythms [15]. The approach using embryonic stem cells carry the problems of identifying appropriate cell lineages, possibility of stem cell differentiation into lines other than pacemaker cells, and potential for neoplasia. Adult mesenchymal stem cells are biologically inert vectors which can deliver genetic information to myocardium. Human mesenchymal stem cells (hMSCs) as a platform for delivery of genes into heart is a more attractive option because they can be obtained in large numbers, easily expanded in culture, capable of long term transgene expression and their administration can be autologous or via banked stores [15].

## Gene therapy versus stem cell therapy

In gene therapy a cardiac myocyte is converted into a pacemaker cell whereas in stem cell therapy myocytes retain their original function. An inherent problem of gene therapy is use of viruses. Replication deficient adenoviruses with little infectious potential lead to only transient improvement in pacemaker function. Retroviruses may be carcinogenic and infective.

## Important studies on biological pacemakers

### 1. Molecular transfer of the human β_2_ Adrenergic receptor cDNA

Effects of transferring the human β_2_ adrenergic receptor were studied by Edelberg JM et al [1] in chronotropy studies with isolated myocytes, and transplanted as well as endogenous murine heart. Murine embryonic cardiac myocytes were transiently transfected with plasmid constructs. The total percentage of spontaneously contracting myocytes was greater in β_2_AR transfected cells compared with controls. Also the percentage of myocytes with chronotropic rates more than 60 beats per minute was greater in β_2_AR population than controls. To study the ex vivo effects of targeted expression of β_2_AR a murine neonatal cardiac transplantation model was used. Injection of β_2_AR construct increased the heart rate by 40%. These studies demonstrate that local targeting of gene expression may be a feasible modality to regulate the cardiac pacemaking activity.

### 2. Local expression of HCN2 in canine left atrium

Research by Jihong Qu et al [13] showed that HCN2 over expression provides an I_f_ - based pacemaker current sufficient to drive the heart when injected into a localized region of atrium. Adenoviral constructs of mouse HCN2 and green fluorescent protein (GFP) or GFP alone were injected into LA, terminal studies performed 3-4 days later, myocytes examined for native and expressed pacemaker current ( I_f_). Spontaneous LA rhythms occurred after vagal stimulation-induced sinus arrest in 4 of 4 HCN2 + GFP dogs and 0 of 3 GFP dogs (P<0.05).

### 3. Biological pacemaker implanted in canine left bundle branch

Alexi N. Plotnikov et al [14] studied the effect of administration of the HCN2 gene to the left bundle branch system of dogs. An adenoviral construct incorporating HCN2 and green fluorescent protein (GFP) as a marker was injected via catheter under fluoroscopic control into the posterior division of the LBB. Controls were injected with an adenoviral construct of GFP alone or saline. During vagal stimulation, HCN2 injected dogs showed rhythms originating from the left ventricle, the rate of which was significantly more rapid than controls.

### 4. Human mesenchymal Stem Cells as a gene delivery system to create cardiac pacemaker

Potapova I et al [3] tested the ability of human mesenchymal stem cells to deliver a biological pacemaker to the heart. hMSCs transfected with a cardiac pacemaker gene, mHCN2, by electroporation expressed current as I_f_- like. They demonstrated that genetically modified hMSCs can express functional HCN2 channels in vitro and in vivo, mimicking over exression of HCN2 genes in cardiac myocytes, and represent a noval delivery system for pacemaker genes into the heart or other electrical syncytia.

## Limitations of approaches to development of biological pacemaker

Use of viruses to deliver the necessary genes has inherent problems. Replication deficient adenoviruses that have little infectious potential lead to only   transient improvement in pacemaker function as well as potential inflammatory responses. Retroviruses carry a risk of carcinogenicity and infectivity. Limitations of stem cell therapy include immunogenicity of cell, the potential for neoplasia, proper engineering of pure cardiac lineages and spatial non uniformity of implants. Regulating the level of expression to achieve optimal pacemaker rate is critical. Biological pacemaker needs an optimal cell mass and optimal cell-cell coupling for long term normal function. Research is ongoing to identify optimal cell numbers and coupling ratios needed to optimize the function of biological pacemakers.

A major issue is duration of efficacy of biological pacemakers. The duration of pacemaker function in approaches using viruses depend on how long the viruses and resulting protein constructs survive in the host. To ensure long term function the appropriate delivery system in which the construct is effective for long periods must be identified. What will be the longevity and stability of next generation of pacemaker genes?

The onset of pacemaker function after a pause following the last intrinsic beat is a critical factor. Can a pacemaker gene inserted into proximal conduction system create a functioning biological pacemaker which can drive the ventricle in demand mode when the sinus node signal fails? This requires proper engineering of genes. Considering the cell-cell coupling differences in gene therapy and stem cell therapy, the engineering of mutant genes will differ importantly between approaches.

The autonomic responsiveness of biological pacemakers, the ideal site for implantation, the extent of recovery of diseased sinus node and the ideal construct to be preferred remain unanswered questions. None of the studies tested whether a biological pacemaker could be engineered into the ventricular conducting system. Will the functional characteristics of biological pacemakers compete with that of electronic units available?
